# Neurophysiological Markers of Adaptation and Compensation Following Lower Limb Amputation: An Analysis of EEG Oscillations and Clinical Predictors from the DEFINE Cohort Study

**DOI:** 10.3390/neurolint17020021

**Published:** 2025-01-28

**Authors:** Guilherme J. M. Lacerda, Valton Costa, Lucas Camargo, Linamara R. Battistella, Marta Imamura, Felipe Fregni

**Affiliations:** 1 Neuromodulation Center and Center for Clinical Research Learning, Spaulding Rehabilitation Hospital and Massachusetts General Hospital, Harvard Medical School, Boston, MA 02115, USA; guilhermelacerda7@gmail.com (G.J.M.L.); valtoncosta@estudante.ufscar.br (V.C.); lcamargo@mgh.harvard.edu (L.C.); 2 Institute of Physical Medicine and Rehabilitation, Faculty of Medicine, University of São Paulo (USP), São Paulo 04116-030, Brazil; linamara@usp.br (L.R.B.); marta.imamura@fm.usp.br (M.I.); 3 Laboratory of Neurosciences and Neurological Rehabilitation, Physical Therapy Department, Federal University of Sao Carlos, Sao Carlos 13565-905, Brazil; 4 Department of Legal Medicine, Bioethics, Occupational Medicine, Physical Medicine and Rehabilitation, Faculty of Medicine, University of Sao Paulo (USP), São Paulo 01246-903, Brazil

**Keywords:** neurophysiological biomarkers, brain oscillations, electroencephalography, amputation, clinical predictors

## Abstract

**Background:** Neuroplasticity, involving cortical and subcortical reorganization, plays a critical role in the adaptation and compensation process post-amputation. However, underlying neurophysiological changes remain unclear, particularly in brain oscillations. **Methods:** This is a cross-sectional analysis that includes baseline data from 48 individuals with lower limb amputation from our DEFINE Cohort Study project. EEG data were collected using a 64-channel system during a 5-min resting-state period. Preprocessed data were analyzed for delta and alpha oscillations across frontal, central, and parietal regions. Logistic regression models examined associations between EEG oscillations and clinical variables, including cognition (MoCA), functional independence (FIM), and phantom limb sensations (PLS). **Results:** The multivariate logistic regression analysis revealed distinct patterns of association between EEG oscillations and clinical variables. Delta oscillations were inversely associated with cognitive scores (OR: 0.69; *p* = 0.048), while higher delta power was related to the absence of PLS (OR: 58.55; *p* < 0.01). Frontal alpha power was positively linked to cognitive function (OR: 1.55; *p* = 0.02) but negatively associated with functional independence (OR: 0.75; *p* = 0.04). **Conclusions:** These findings suggest that lower frequencies, such as delta oscillations, play a role as potential compensatory brain rhythms. In contrast, alpha oscillations may reflect a more adapted pattern of brain reorganization after amputation.

## 1. Introduction

Limb loss is a significant cause of morbidity and economic burden, affecting patients and healthcare systems worldwide. The primary causes of lower limb amputation include trauma, metabolic conditions, and vascular diseases, such as diabetes mellitus and peripheral arterial disease [1]. From 2008 to 2020, Brazil recorded over 600,000 lower limb amputations [2]. In the United States, approximately 120,000 lower limb amputations are performed each year, with the lifetime healthcare cost per individual estimated at around USD 650,000 in 2013 [3].

Amputation significantly reduces quality of life, often resulting in decreased work productivity, increased susceptibility to further health complications, and a higher mortality risk than the general population [4]. Additionally, most amputees develop phantom limb pain (PLP), with studies reporting a prevalence of 70–80% [5,6]. Despite the high prevalence and impact of PLP and other amputation-related complications, providing effective care for this population remains challenging, as the mechanisms underlying structural brain reorganization following limb amputation are not yet fully understood.

Following an upper or lower limb amputation, neuroplasticity processes are activated to adapt cortical and subcortical regions to the absence of the limb. This reorganization involves several circuits, including cortical, corticothalamic, and limbic pathways associated with motor execution, somatosensory function, nociception, and spatial awareness [7,8,9,10,11]. Neuroimaging studies in both animals and humans have shown structural remodeling in cortical and subcortical areas following limb loss [12,13,14,15]. Due to its low cost and high temporal resolution, electroencephalography (EEG) is widely used to quantify cortical activity changes in patients across various medical conditions [16].

In our previous research based on the DEFINE cohort [17] and other projects, we investigated resting-state EEG oscillations as potential biomarkers of adaptive and compensatory mechanisms linked to clinical recovery and rehabilitation outcomes across multiple conditions, including chronic neuropathic pain, fibromyalgia, stroke, KOA, and NSLBP [18,19,20,21,22,23,24,25].

One of these studies, focused on chronic neuropathic pain, identified delta and theta bands as compensatory oscillations negatively associated with pain level. In contrast, higher frequencies, particularly in the alpha range, were linked to disruptive mechanisms, such as pain and mood impairment related to pain [18]. In fibromyalgia, increased theta power was associated with a greater likelihood of memory complaints and other clinical features, including older age, sleep problems, and anxiety, suggesting a compensatory role for the theta band.

In stroke, delta and beta power increases were proposed as markers of maladaptive brain plasticity associated with poorer functional outcomes. By contrast, increased theta and alpha power were linked to better sensorimotor outcomes, indicating these bands as compensatory oscillations [26]. In knee osteoarthritis (KOA), findings showed a positive association between fronto-central alpha and pain intensity, alongside a negative association between theta activity and pain intensity [20]. These results suggested that elevated alpha over sensorimotor areas may reflect a maladaptive compensatory response to impaired motor function and advanced joint degeneration, thus highlighting distinct KOA phenotypes [20]. On the other hand, the presence of physical disability and chronic pain can lead to chronic distress, inflammation, and downregulation of neural activity in the hippocampus, resulting in lower cognitive performances, like working memory, often associated with EEG low-frequency bands [25]. Moreover, diffuse delta and theta power were positively associated with poor cognition, aging, and depressive symptoms [20]. 

These studies are essential for understanding the mechanisms underlying pathological, compensatory, and adaptive processes in these health conditions, and they may help identify biomarkers to improve healthcare and management strategies. However, neurophysiological research into structural and functional changes after amputations and other neurological conditions still yields varied results given the diversity of conditions and metrics involved. In this exploratory study, we examine the predictive value of clinical and demographic factors on brain resting-state oscillations, assessing their potential as neurophysiological markers of adaptation and compensation following lower limb amputation.

## 2. Materials and Methods

### 2.1. Participants, Study Design, and Sample Size

This is an exploratory cross-sectional analysis that includes baseline data from 48 individuals with lower limb amputation from our DEFINE Cohort Study project [17]. All participants were admitted to the Instituto de Medicina Física e Reabilitação (IMREA) at the University of Sao Paulo (USP), Brazil. Patients with a previous history of neurological conditions such as stroke, epilepsy, dementia, brain injury, or psychiatric conditions were excluded. This project was approved by the Ethics Committee of the Hospital das Clínicas, Faculty of Medicine of the University of Sao Paulo. All participants provided informed consent following the Declaration of Helsinki (1964) [27].

### 2.2. Demographic and Clinical Variables

Demographic and medical history data were collected from participants, including age, sex assigned at birth, race, educational level, body mass index (BMI), the cause of amputation, and the presence and frequency of phantom limb sensation (PLS) and other phantom phenomena as evaluated in the Groningen Questionnaire Problems After Leg Amputation. Additionally, validated scales were used to assess functional and cognitive status, including the Functional Independence Measure (FIM), Montreal Cognitive Assessment (MoCA), and the Visual Analog Scale (VAS) for pain. Further details on these instruments can be found in our cohort study protocol [17].

### 2.3. Electroencephalography (EEG)

EEG data were collected using two systems: the ANT Neuro 64-channel EEG system (ANT Neuro, Enschede, The Netherlands) and the Brain Vision ActiCHamp 64-channel EEG system (Brain Products GmbH, Gilching, Germany) during a 5-min eyes-closed resting-state period. The data were then exported and analyzed offline using MATLAB (R2014b, The MathWorks Inc., Natick, MA, USA) and EEGLab.

The preprocessing pipeline included the following steps: (i) bandpass filtering with a high-pass filter at 1 Hz and a low-pass filter at 50 Hz, (ii) downsampling from 1000 Hz to 250 Hz, (iii) re-referencing the channels using the average of all electrodes, and (iv) 60 Hz power line noise correction (specific to Brazil). 

An expert clinical neurophysiologist visually inspected the EEG data to identify artifacts and any potential clinical abnormalities before performing an Independent Component Analysis (ICA). Channels were removed if they met any of the following criteria: (i) flat for longer than three seconds, (ii) exhibiting high-frequency noise exceeding two standard deviations, or (iii) showing a correlation with neighboring channels of less than 0.8, as assessed using the Clean_rawdata EEGLAB plugin (v2.2). The remaining channels were then processed using the Infomax ICA algorithm, implemented with the Darbeliai plugin, identifying and removing artifacts [28,29]. ICA algorithms are particularly effective at eliminating noise signals from heart rate, muscle activity, blinking, and eye movements [30].

The following standard frequency bands were analyzed: delta (1–3.9 Hz), theta (4–7.9 Hz), low-alpha (8–9.9 Hz), high-alpha (10–12.9 Hz), alpha (8–12.9 Hz), low-beta 1 (13–19.9 Hz), high-beta 2 (20–30 Hz), and beta (13–30 Hz). These bands were analyzed in the following regions of interest (ROIs): frontal (F3, F4, F7, F8, FCz, FP1, FP2, Fz), central (C3, C4, CP1, CP2, CP5, CP6, Cz), and parietal (O1, O2, Oz, P3, P4, Pz) areas. 

### 2.4. Statistical Analysis

Our analysis followed several steps. First, because the EEG oscillation data were not normally distributed, we converted them into binary categories based on the median value for each oscillation. Logistic regression was then used to model the relationships between these binary outcomes and the independent variables. Univariate logistic regressions were conducted against all demographic and clinical variables for each EEG oscillation. Variables with a univariate *p*-value < 0.25 and those considered clinically meaningful were subsequently included in the multivariate models. No adjustments for multiple comparisons were made to maximize sensitivity in this exploratory study. Statistical analyses were performed using RStudio (Version 2023.06.0 + 421). Results were considered statistically significant at *p* < 0.05. 

## 3. Results

### 3.1. Sample Characteristics

Baseline data were collected from 48 participants included in the study, with a median age of 49 years (IQR: 25.5). The sample was predominantly male (40 individuals, 83%) and included a smaller proportion of females (8 individuals, 17%). Most amputations were above the knee (65%, 31 participants), while 35% were below the knee (17 participants). The median duration since amputation was 21.4 months (IQR: 21.7). A detailed characterization of the sample, including relevant clinical and demographic features, is provided in Table 1. 

### 3.2. Description of Dependent Variables

EEG data were collected from 48 participants. Table 2 presents brain oscillations, averaged across both hemispheres, revealing distinct patterns across different regions. Delta oscillations in the central region had a median value of 0.14 (IQR: 0.11), while alpha oscillations in the frontal region exhibited a higher median value of 0.51 (IQR: 0.29).

### 3.3. Univariate Analysis

We first conducted univariate logistic regression to identify variables associated with EEG oscillations. This analysis revealed statistically significant relationships between each oscillation and various independent variables.

### 3.4. Multivariate Analysis

Subsequently, multivariate logistic regression models were constructed using a forward selection technique. Variables were added sequentially based on either univariate significance (*p* < 0.25) or their clinical relevance to the study. This approach allowed us to investigate the relationship between EEG oscillations and clinical variables, assessing whether these oscillations could be linked to compensatory system activity in this patient group. Age was not a significant factor in any model. The variables finally included for each model can be seen in Table 3.

### 3.5. Delta Oscillations

For delta oscillations, we observed significant associations with both MOCA scores and the presence of PLS (*n* = 31). Specifically, there was a negative association with MOCA scores, where each unit increase in the MOCA was associated with a decrease in the odds of delta oscillations (OR = 0.69; 95% CI = 0.43–0.94; *p* = 0.048). In contrast, a strong positive association was found with PLS, with patients reporting PLS having significantly higher odds of delta oscillations (OR = 58.55; 95% CI = 6.21–1762; *p* = 0.003). The model’s McFadden’s R^2^ was 0.46.

### 3.6. Alpha Oscillations

Significant associations were identified with both MOCA and FIM scores for alpha oscillations (*n* = 27). A positive association was observed with the MOCA, where each unit increase in the MOCA was linked to higher odds of alpha oscillations (OR = 1.55; 95% CI = 1.13–2.47; *p* = 0.022). Conversely, FIM scores showed a negative association, with higher FIM scores corresponding to lower odds of alpha oscillations (OR = 0.75; 95% CI = 0.52–0.92; *p* = 0.041). The model’s McFadden’s R^2^ was 0.28.

## 4. Discussion

This study explored the association between sociodemographic, clinical variables, and resting-state EEG spectral power in individuals with lower limb amputations, addressing a gap in the current literature. Following limb amputation, neural reorganization processes, such as cortical remapping, are expected to occur in sensorimotor cortices and other cortical regions, including interhemispheric changes [7,8,31]. Additionally, these changes may be influenced by an individual’s ability to adapt to their new situation, whether positively or negatively. For example, some individuals develop chronic phantom and residual limb pain, and related emotional and cognitive comorbidities, while others adjust functionally and pain-free [7,32]. Thus, changes in brain oscillations may reflect this adaptation process. Our findings suggest that delta and alpha oscillations showed cortical reorganization in individuals with lower limb amputation, which could be crucial in the compensatory adjustments within the brain’s networks during their medical rehabilitation. These results provide relevant insights to be deeply investigated in future longitudinal studies. In this study, we built two multivariate models to investigate delta and alpha EEG oscillations in the frontal and central brain areas and their relationships with cognitive, functional, and clinical predictors.

### 4.1. Central Delta Oscillations Model

Our model revealed significant associations between cognition, the presence of phantom limb sensation (PLS), and central delta power. Specifically, better cognitive function was associated with lower delta power, while the absence of PLS was strongly associated with higher delta power. A previous study suggested that delta oscillations could serve as compensatory markers, as they were found to correlate with lower levels of chronic neuropathic pain [18]. In this context, higher delta power may indicate a more effective compensatory response. However, in our study, pain was not associated with delta power. Instead, increased delta power was observed in participants without PLS. PLS has been linked to positive adaptation after limb amputation, where intense non-painful PLS, especially phantom limb movement, is associated with less pain and better adaptation, likely reflecting compensatory mechanisms in sensorimotor networks [33,34]. This interpretation is reinforced by the results of three previous studies showing that a decreased central delta power was associated with better motor function in stroke and lower levels of pain in KOA and NSLBP [19,23,26,35].

PLS can encompass a plethora of phantom manifestations, including electric, itching, and touching; some can be very intense and disturbing, even when not felt as a painful sensation [33]. In this sense, an alternative interpretation is that the absence of PLS could represent a positive sign of adaptation, supporting higher delta power as a marker of better compensatory response, consistent with findings in chronic neuropathic pain. On the other hand, stroke and limb amputation share mechanisms of hemispheric impairment and interhemispheric imbalances, which may underlie similar reorganization and sensorimotor adaptation processes. Thus, decreased delta power could be interpreted as a marker of better adaptation.

Cognitive function is a critical factor after amputation, influencing mobility and participation in post-amputation rehabilitation. For instance, cognition has been associated with mobility, daily living activities, and prosthetic use in individuals with non-traumatic lower limb amputations [36,37]. Additionally, cognitive function and delta power in the central, frontal, and parietal areas have been linked to conditions such as KOA and stroke [20]. Delta oscillations, reflecting corticothalamic interactions, have been associated with homeostatic sleep drive, sensory processing, and aging [20,38,39]. The limb loss can still have an important representation in the motor and sensory cortical areas even many years after the amputation, which often leads to an overreaction of low-frequency bands in the presence of PLP in M1 and S1 [7,40,41]. Therefore, in the context of the amputee population, a reduction in delta oscillations seems to indicate neuroplasticity processes and better clinical outcomes. Additionally, decreases in delta power have also been linked to improved cognitive therapy outcomes in sleep medicine, while increased delta power has been associated with poorer recovery post-stroke [38,42]. Our findings suggest that central delta power could serve as a potential biomarker for adaptation and functional recovery after amputation.

### 4.2. Frontal Alpha Oscillations Model

This model shows a positive association between cognitive function and frontal alpha power. On the other hand, it shows a negative association between functional independence and alpha power in the frontal area. Alpha oscillations are related to different brain functions such as cognitive processing, sensorimotor regulation, and emotional and pain control [43,44,45]. Our results are in line with previous studies that observed a positive relationship between cognition and alpha oscillations in both healthy adults and those with cognitive decline [45,46,47,48].

The negative association with functional independence, however, warrants further consideration. In our prior studies, divergent associations were observed depending on the condition. For instance, high alpha power was positively associated with pain and impaired mood in chronic neuropathic pain and with pain and stiffness in KOA [18,20]; however, in stroke, it was positively associated with sensorimotor outcomes and negatively associated with depression [26]. Alpha oscillations are modulated during movement preparation and are suppressed by sensorimotor activity. Consequently, higher resting alpha power might reflect overactive sensorimotor networks, while lower alpha power at rest may indicate better-adjusted sensorimotor networks, similar to what is observed after motor learning [49]. This could explain the negative relationship regarding functional independence observed in our model. In fact, changes in the functional network in the alpha band have been reported after amputation, suggesting increased neural synchronization [50]. It was hypothesized that these changes may reflect the unmasking and strengthening of silent or previously subthreshold connections at local and network levels and likely relate to reported phantom limb perception or phantom limb pain, which could interfere with motor performance and functional outcomes. However, the observed relationship should be seen with caution as we identified slight variance in functional independence in our sample, with most of it showing high levels of autonomy. Therefore, the odds ratio for this predictor may be less reliable or inflated due to a potential ceiling effect.

### 4.3. Limitations and Future Directions

The main limitations of our study are related to the exploratory cross-sectional design, the absence of a control group, and the sample size. The small sample reduced statistical power, particularly when adjusting for multiple variables. Some adjustments would be of great importance. For example, in the central delta power model, adjusting for factors like time since amputation, sleep quality, sex, and age would have been valuable but was not possible due to sample constraints.

Future studies could solve these limitations by extending the sample size and the number of observations and testing some of the raised hypotheses in longitudinal studies to observe the change in brain reorganization over time. For example, it can reproduce the models and test the sensibility of the delta and alpha oscillations to detect changes in cognition, motor function, and other metrics of neural adaptation over time from pre- to post-amputation. Crucially, mechanistic studies must investigate the role of these EEG markers across different health conditions to clarify if the changes in these oscillations serve as general markers of brain adaptation and compensatory response or reflect unique condition- or conditions-specific pathways.

## 5. Conclusions

This study provides novel insights into the relationship between resting-state EEG oscillations and clinical, cognitive, and functional variables in individuals with lower limb amputations. We identified distinct associations between delta and alpha oscillations and factors such as cognitive function and phantom limb sensation. Our findings suggest that EEG oscillations may be biomarkers of neural adaptation and compensatory responses following amputation in the delta and alpha bands. Specifically, increased delta power in the absence of phantom limb sensation may indicate positive neural adaptation. In contrast, frontal alpha power could reflect the balance between sensorimotor network regulation and functional independence.

Although this study’s exploratory nature and sample size limit generalizability, these findings open avenues for future research. More extensive, longitudinal studies are needed to confirm the sensitivity of EEG oscillations as markers of functional recovery and neural adaptation in this population. We believe that EEG oscillations during resting state can indicate cortical reorganization and are potential tools to help clinicians with the diagnosis and evaluate treatment responses in the medical rehabilitation field related to phantom limb pain and physical and cognitive functions. Understanding the mechanistic role of these oscillations across different conditions will be crucial for refining rehabilitation strategies and improving outcomes for individuals with amputations.

## Figures and Tables

**Table 1:** Characterization of the sample (*n* = 48).

Variable	Median (IQR) or *n* (%)
Age	49 (25.5)
Sex	
Male	40 (83%)
Female	8 (17%)
Race	
White	30 (63%)
Non-white	18 (37%)
Civil status	
Single	18 (37%)
Married	21 (43%)
Divorced	7 (15%)
Widowed	2 (5%)
Amputation side	
Right	18 (37%)
Left	28 (59%)
Bilateral	2 (4%)
Amputation level	
Above the knee	31 (65%)
Below the knee	17 (35%)
BMI	24.6 (5.1)
Amputation duration (months)	21.4 (21.7)
Education (years)	10 (6)
FIM	117 (5.25)
MOCA	22 (6)
PLS	
Presence	34 (70%)
Absence	14 (30%)

BMI: body mass index; FIM: Functional Independence Measure; MOCA: Montreal Cognitive Assessment; PLS: phantom limb sensation.

**Table 2:** Descriptive data of the dependent variables (brain oscillations), *n* = 48.

Variable	Median (IQR)
Delta Oscillations	
Central Region	0.14 (0.11)
Alpha Oscillations	
Frontal Region	0.51 (0.29)

**Table 3:** Multivariate logistic regression models.

Models	OR (95% CI)	Std. Error	*p*-Value	t-Stat	AUC
Delta Oscillations					0.93
MOCA	0.69 (0.43–0.94)	0.05	0.048	3.74	
PLS	58.55 (6.21–1762)	1.37	0.003	42.8	
Alpha Oscillations					0.82
MOCA	1.55 (1.13–2.47)	0.19	0.022	8.08	
FIM	0.75 (0.52–0.92)	0.14	0.041	5.22	

MOCA: Montreal Cognitive Assessment; PLS: phantom limb sensation; FIM: Functional Independence Measure. AUC: area under the curve.

## Data Availability

The data that support the findings of this study are available from the corresponding author upon reasonable request due to privacy reasons.

## References

[1] Hussain M.A., Al-Omran M., Salata K., Sivaswamy A., Forbes T.L., Sattar N., Aljabri B., Kayssi A., Verma S., Mestral C.D. (2019). Population-based secular trends in lower-extremity amputation for diabetes and peripheral artery disease. CMAJ.

[2] Portela F.S.O., Louzada A.C.S., Silva M.F.A.D., Teivelis M.P., Kuzniec S., Wolosker N. (2024). Editor’s Choice—Analysis of Lower Limb Amputations in Brazil’s Public Health System over 13 Years. Eur. J. Vasc. Endovasc. Surg..

[3] Xu J., Haider A., Sheikh A., González-Fernández M. (2024). Epidemiology and Impact of Limb Loss in the United States and Globally. Phys. Med. Rehabil. Clin. N. Am..

[4] Ma V.Y., Chan L., Carruthers L.J. (2014). The Incidence, Prevalence, Costs and Impact on Disability of Common Conditions Requiring Rehabilitation in the US: Stroke, Spinal Cord Injury, Traumatic Brain Injury, Multiple Sclerosis, Osteoarthritis, Rheumatoid Arthritis, Limb Loss, and Back Pain. Arch. Phys. Med. Rehabil..

[5] Limakatso K., Ndhlovu F., Usenbo A., Rayamajhi S., Kloppers C., Parker R. (2024). The prevalence and risk factors for phantom limb pain: A cross-sectional survey. BMC. Neurol..

[6] Hulsey D.R., Hays S.A., Khodaparast N., Ruiz A., Das P., Rennaker R.L., Kilgard M.P. (2016). Reorganization of Motor Cortex by Vagus Nerve Stimulation Requires Cholinergic Innervation. Brain Stimul..

[7] Makin T.R., Flor H. (2020). Brain (re)organisation following amputation: Implications for phantom limb pain. Neuroimage.

[8] Bao B.-B., Zhu H.-Y., Wei H.-F., Li J., Wang Z.-B., Li Y.-H., Hua X.-Y., Zheng M.X., Zheng X.-Y. (2022). Altered intra- and inter-network brain functional connectivity in upper-limb amputees revealed through independent component analysis. Neural. Regen. Res..

[9] Chen M., Yu L., Ouyang F., Liu Q., Wang Z., Wang S., Zhou L., Jiang H., Zhou S. (2015). The right side or left side of noninvasive transcutaneous vagus nerve stimulation: Based on conventional wisdom or scientific evidence?. Int. J. Cardiol..

[10] Pacheco-Barrios K., Pinto C.B., Velez F.G.S., Duarte D., Gunduz M.E., Simis M., Gianlorenco A.C.L., Barouh J.L., Crandell M., Battistella L. (2020). Structural and functional motor cortex asymmetry in unilateral lower limb amputation with phantom limb pain. Clin. Neurophysiol..

[11] Bramati I.E., Rodrigues E.C., Simões E.L., Melo B., Höfle S., Moll J., Lent R., Tovar-Moll F. (2019). Lower limb amputees undergo long-distance plasticity in sensorimotor functional connectivity. Sci. Rep..

[12] Florence S.L., Kaas J.H. (1995). Large-scale reorganization at multiple levels of the somatosensory pathway follows therapeutic amputation of the hand in monkeys. J. Neurosci..

[13] Zhang H., Zhou Q.-Q., Chen H., Hu X.-Q., Li W.-G., Bai Y., Han J.-X., Wang Y., Liang Z.-H., Chen D. (2023). The applied principles of EEG analysis methods in neuroscience and clinical neurology. Mil. Med. Res..

[14] Jian N., Florence S.L., Qi H.X., Kaas J.H. (2000). Growth of new brainstem connections in adult monkeys with massive sensory loss. Proc. Natl. Acad. Sci. USA.

[15] Draganski B., Moser T., Lummel N., Gänssbauer S., Bogdahn U., Haas F., May A. (2006). Decrease of thalamic gray matter following limb amputation. Neuroimage.

[16] Hughes J.R., John E.R. (1999). Conventional and quantitative electroencephalography in psychiatry. J. Neuropsychiatry Clin. Neurosci..

[17] Simis M., Imamura M., Melo P.S.D., Marduy A., Battistella L., Fregni F. (2021). Deficit of Inhibition as a Marker of Neuroplasticity (DEFINE Study) in Rehabilitation: A Longitudinal Cohort Study Protocol. Front. Neurol..

[18] Barbosa S.P., Junqueira Y.N., Akamatsu M.A., Marques L.M., Teixeira A., Lobo M., Mahmoud M.H., Omer W.E., Pacheco-Barrios K., Fregni F. (2024). Resting-state electroencephalography delta and theta bands as compensatory oscillations in chronic neuropathic pain: A secondary data analysis. Brain. Netw. Modul..

[19] Marques L.M., Barbosa S.P., Pacheco-Barrios K., Goncalves F.T., Imamura M., Battistella L.R., Simis M., Fregni F. (2022). Motor event-related synchronization as an inhibitory biomarker of pain severity, sensitivity, and chronicity in patients with knee osteoarthritis. Neurophysiol. Clin..

[20] Simis M., Imamura M., Pacheco-Barrios K., Marduy A., Melo P.S.D., Mendes A.J., Teixeira P.E.P., Battistella L., Fregni F. (2022). EEG theta and beta bands as brain oscillations for different knee osteoarthritis phenotypes according to disease severity. Sci. Rep..

[21] Simis M., Camsari D.D., Imamura M., Filippo T.R.M., Souza R.D., Battistella L.R., Fregni F. (2021). Electroencephalography as a Biomarker for Functional Recovery in Spinal Cord Injury Patients. Front. Hum. Neurosci..

[22] Zortea M., Beltran G., Alves R.L., Vicuña P., Torres I.L.S., Fregni F. (2021). Spectral Power Density analysis of the resting-state as a marker of the central effects of opioid use in fibromyalgia. Sci. Rep..

[23] Teixeira M., Mancini C., Wicht C.A., Maestretti G., Kuntzer T., Cazzoli D., Mouthon M., Annoni J.-M., Chabwine J.N. (2021). Beta Electroencephalographic Oscillation Is a Potential GABAergic Biomarker of Chronic Peripheral Neuropathic Pain. Front. Neurosci..

[24] Camargo L., Pacheco-Barrios K., Marques L.M., Caumo M., Fregni F. (2024). Adaptive and Compensatory Neural Signatures in Fibromyalgia: An Analysis of Resting-State and Stimulus-Evoked EEG Oscillations. Biomedicines.

[25] Pacheco-Barrios K., Teixeira P.E.P., Martinez-Magallanes D., Neto M.S., Pichardo E.A., Camargo L., Lima D., Cardenas-Rojas A., Fregni F. (2024). Brain compensatory mechanisms in depression and memory complaints in fibromyalgia: The role of theta oscillatory activity. Pain. Med..

[26] Marques L.M., Barbosa S.P., Gianlorenço A.C., Pacheco-Barrios K., Souza D.R., Matheus D., Battistella L., Simis M., Fregni F. (2024). Resting-state EEG as Biomarker of Maladaptive Motor Function and Depressive Profile in Stroke Patients. Clin. EEG. Neurosci..

[27] Rickham P.P. (1964). Human Experimentation. Code of Ethics of the World Medical Association. Declaration of Helsinki. Br. Med. J..

[28] Delorme A., Makeig S. (2004). EEGLAB: An open source toolbox for analysis of single-trial EEG dynamics including independent component analysis. J. Neurosci. Methods.

[29] Delorme A., Sejnowski T., Makeig S. (2007). Enhanced detection of artifacts in EEG data using higher-order statistics and independent component analysis. Neuroimage.

[30] Pion-Tonachini L., Kreutz-Delgado K., Makeig S. (2019). ICLabel: An automated electroencephalographic independent component classifier, dataset, and website. Neuroimage.

[31] Jiang G., Yin X., Li C., Li L., Zhao L., Evans A.C., Jiang T., Wu J., Wang J. (2015). The Plasticity of Brain Gray Matter and White Matter following Lower Limb Amputation. Neural. Plast..

[32] Sparling T., Iyer L., Pasquina P., Petrus E. (2024). Cortical Reorganization after Limb Loss: Bridging the Gap between Basic Science and Clinical Recovery. J. Neurosci..

[33] Münger M., Pinto C.B., Pacheco-Barrios K., Duarte D., Gunduz M.E., Simis M., Battistella L.R., Fregni F. (2020). Protective and Risk Factors for Phantom Limb Pain and Residual Limb Pain Severity. Pain. Pract..

[34] Ortega-Márquez J., Garnier J., Mena L., Palagi A.V., Grützmacher E.B., Vallejos-Penaloza G., Costa V., Martinez-Magallanes D., Macedo A.V.D., Fregni F. (2024). Clinical Characteristics Associated with the PLP-PLS Index, a New Potential Metric to Phenotype Phantom Limb Pain. Biomedicines.

[35] Marques L.M., Castellani A., Barbosa S.P., Imamura M., Battistella L.R., Simis M., Fregni F. (2024). Neuroplasticity changes in knee osteoarthritis (KOA) indexed by event-related desynchronization/synchronization during a motor inhibition task. Somatosens Mot. Res..

[36] Dawes E., Hewitt L.L., Bliokas V.V., Wilson V.J. (2023). A Systematic Review of Cognitive Functioning and its Relationship to Outcomes Following Amputation Secondary to Vascular Etiology. Int. J. Low. Extrem Wounds.

[37] Lee D.J., Costello M.C. (2018). The effect of cognitive impairment on prosthesis use in older adults who underwent amputation due to vascular-related etiology: A systematic review of the literature. Prosthet Orthot Int..

[38] Long S., Ding R., Wang J., Yu Y., Lu J., Yao D. (2021). Sleep Quality and Electroencephalogram Delta Power. Front Neurosci..

[39] Gunasekaran H., Azizi L., Wassenhove V.V., Herbst S.K. (2023). Characterizing endogenous delta oscillations in human MEG. Sci. Rep..

[40] MacIver K., Lloyd D.M., Kelly S., Roberts N., Nurmikko T. (2008). Phantom limb pain, cortical reorganization and the therapeutic effect of mental imagery. Brain.

[41] Liu S., Fu W., Wei C., Ma F., Cui N., Shan X., Zhang Y. (2022). Interference of unilateral lower limb amputation on motor imagery rhythm and remodeling of sensorimotor areas. Front. Hum. Neurosci. Orig. Res..

[42] Cassidy J.M., Wodeyar A., Wu J., Kaur K., Masuda A.K., Srinivasan R., Cramer S.C. (2020). Low-Frequency Oscillations Are a Biomarker of Injury and Recovery After Stroke. Stroke.

[43] Thomaidou M.A., Blythe J.S., Houtman S.J., Veldhuijzen D.S., Laarhoven A.I.M.V., Evers A.W.M. (2021). Temporal structure of brain oscillations predicts learned nocebo responses to pain. Sci. Rep..

[44] Babiloni C., Percio C.D., Arendt-Nielsen L., Soricelli A., Romani G.L., Rossini P.M., Capotosto P. (2014). Cortical EEG alpha rhythms reflect task-specific somatosensory and motor interactions in humans. Clin. Neurophysiol..

[45] Melo H.M.N., Martins L., Oldemburgo M.V. (2017). Takase, Emílio, Influência do ritmo Alfa (8-12Hz) no tempo de reação em uma tarefa de controle inibitório. Neuropsicol. Latinoam..

[46] Brickwedde M., Krüger M.C., Dinse H.R. (2019). Somatosensory alpha oscillations gate perceptual learning efficiency. Nat. Commu..

[47] Luft C.D.B., Zioga I., Thompson N.M., Banissy M.J., Bhattacharya J. (2018). Right temporal alpha oscillations as a neural mechanism for inhibiting obvious associations. Proc. Natl. Acad. Sci. USA.

[48] Lejko N., Larabi D.I., Herrmann C.S., Aleman A., Ćurčić-Blake B. (2020). Alpha Power and Functional Connectivity in Cognitive Decline: A Systematic Review and Meta-Analysis. J. Alzheimers. Dis..

[49] Gallicchio G., Cooke A., Ring C. (2017). Practice Makes Efficient: Cortical Alpha Oscillations Are Associated With Improved Golf Putting Performance. Sport Exerc. Perform. Psychol..

[50] Lyu Y., Guo X., Wang Z., Tong S. (2016). Resting-state EEG network change in alpha and beta bands after upper limb amputation. Annu. Int. Conf. IEEE. Eng. Med. Biol. Soc..

